# Convergent validity of EQ-5D with core outcomes in dementia: a systematic review

**DOI:** 10.1186/s12955-022-02062-1

**Published:** 2022-11-19

**Authors:** Hannah Hussain, Anju Keetharuth, Donna Rowen, Allan Wailoo

**Affiliations:** School of Health and Related Research, University of Sheffield, 30 Regent St, Sheffield, S1 4DA UK

**Keywords:** Dementia, EQ-5D, Quality of life, Psychometrics, Systematic literature review

## Abstract

**Objectives:**

To explore through a systematic review, the convergent validity of EQ-5D (EQ-5D-3L and EQ-5D-5L (total score and dimensions)) with core outcomes in dementia and investigate how this may be impacted by rater-type; with the aim of informing researchers when choosing measures to use in dementia trials.

**Methods:**

To identify articles relevant to the convergent validity of EQ-5D with core dementia outcomes, three databases were electronically searched to September 2022. Studies were considered eligible for inclusion within the review if they included individual level data from people with dementia of any type, collected self and/or proxy reported EQ-5D and collected at least one core dementia outcome measure. Relevant data such as study sample size, stage of dementia and administration of EQ-5D was extracted, and a narrative synthesis was adopted.

**Results:**

The search strategy retrieved 271 unique records, of which 30 met the inclusion criteria for the review. Twelve different core outcome measures were used to capture dementia outcomes: *cognition, function,* and *behaviour/mood* across the studies. Most studies used EQ-5D-3L (*n* = 27). Evidence related to the relationship between EQ-5D and measures of function and behaviour/mood was the most robust, with unanimous directions of associations, and more statistically significant findings. EQ-5D dimensions exhibited associations with corresponding clinical outcomes, whereby relationships were stronger with proxy-EQ-5D (than self-report).

**Conclusion:**

Measuring health-rated quality of life in dementia populations is a complex issue, particularly when considering balancing the challenges associated with both self and proxy report. Published evidence indicates that EQ-5D shows evidence of convergent validity with the key dementia outcomes, therefore capturing these relevant dementia outcomes. The degree of associations with clinical measures was stronger when considering proxy-reported EQ-5D and differed by EQ-5D dimension type. This review has revealed that, despite the limited targeted psychometric evidence pool and reliance on clinical and observational studies, EQ-5D exhibits convergent validity with other dementia outcome measures.

**Supplementary Information:**

The online version contains supplementary material available at 10.1186/s12955-022-02062-1.

## Background

Dementia is a neurodegenerative condition which mostly affects older adults and is typically characterised by cognitive symptoms such as memory loss and speech and language impairments – but also impacts behaviour, function [1], and general quality of life (QoL) [2]. As the number of people living with dementia is increasing, dementia presents as one of the largest current health and social care challenges facing policymakers [3], whereby evidence regarding the cost-effectiveness of different dementia interventions is fundamental in shaping policy decisions. In the UK, the National Institute for Health and Care Excellence (NICE) recommends the use of EQ-5D to measure health benefit in cost-effectiveness analyses [4]. EQ-5D has five dimensions: mobility; self-care; usual activities; pain/discomfort; and anxiety depression. The EQ-5D-3L has three response categories for each dimension (no problems, some problems, extreme problems) [5] whereas the newer EQ-5D-5L has five response categories (no problems; slight problems; some problems; severe problems; extreme problems/unable to do) [6]. NICE guidelines recommend EQ-5D to measure health related quality of life (HRQoL) as it is a generic measure that enables comparability across disease areas [4, 5].

For decision-making to be optimal, the outcome measure used to measure HRQoL should be responsive and representative of the population in question, and the greatest comparability is produced when the same measure is used across studies. Previous reviews concluded that EQ-5D demonstrated strong acceptability and feasibility within this population due to its concise nature, and that the three-level version displays good overall psychometric properties in dementia research settings [7, 8]. Given that EQ-5D is widely used, is acceptable in dementia populations and recommended by NICE guidelines [5], this systematic review will focus on the EQ-5D measure. There are additional measures that are considered core for collection in dementia studies as they distinctly measure the impact of the key dementia outcomes of:* cognition, function, and behaviour/mood* [1], and therefore together reflect dementia experience (see Table 1). Evidence regarding convergent validity of EQ-5D with these core outcome measures would address the question of how well EQ-5D captures dementia experience and therefore dementia-HRQoL.

**Table 1:** Pre-defined core dementia outcome measures

**Outcome**	**Measurement instrument**
Cognition	MoCA, MMSE, ADAS-Cog, SIB
Activities of daily living	BADLS, DAD, ADCS-ADL, Lawton, Katz Index, Barthel Index
Health-related quality of life	EQ-5D
Behaviour and mood	NPI, CMAI, CSDD, GDS-15

*Notes*: *ADAS-Cog* The Alzheimer's Disease Assessment Scale-Cognitive Subscale, *ADCS-ADL* Alzheimer’s Disease Cooperative Study Activities of Daily Living Scale, *BADLS* Bristol Activities of Daily Living Scale, *CMAI* Cohen-Mansfield Agitation Inventory, *CSDD* Cornell Scale for Depression in Dementia, *DAD* Disability Assessment for Dementia, *GDS-15* Geriatric Depression Scale 15 items, *MoCA* Montreal Cognitive Assessment, *MMSE* Mini-Mental State Examination, *NPI* Neuropsychiatric Inventory, *QoL-AD* Quality of Life in Alzheimer's disease scale, *QUALID* Quality of Life in Late-Stage Dementia Scale*, QUALIDEM* Quality of Life in Late-Stage Dementia Scale proxy*, SIB* Severe Impairment Battery

Two previous recent systematic reviews that have explored HRQoL in dementia have commonly concluded that EQ-5D was the most appropriate utility instrument for use in dementia populations [7, 8]. Although these reviews highlight the value of EQ-5D in dementia research, both reviews broadly investigated psychometric properties, as opposed to specifically focusing on the convergent validity with dementia clinical trial outcomes, which is an important consideration as EQ-5D is increasingly used in dementia populations. A recent review (2022) broadly assessed the psychometric performance of EQ-5D in dementia populations, however it focused solely on EQ-5D-5L [9]. An earlier review (2011) [10] directly explored EQ-5D-3L as a QoL measure in people with dementia (PwD), investigating various psychometric properties including feasibility, reliability, responsiveness and validity. However, the authors highlighted a key recurring theme in the lack of association between self-rated and proxy-rated EQ-5D, indicating problems with inter-rater reliability [10].

Proxy-report is when someone is asked to report on behalf of someone else, typically performed by a family member, caregiver, or healthcare professional. Proxy-reports are particularly important in dementia research as there are specific challenges associated with collecting outcomes within a population of deteriorating cognition, including impaired recall and judgement [11]. It is established within the literature that HRQoL reports made by PwD and proxies do not align, with self-reports often reflecting more optimistic responses [2, 10, 12–14]. This divergence may be more pronounced for some aspects of HRQoL than others. Certain dimensions of HRQoL i.e., anxiety/depression and pain/discomfort in EQ-5D may be “less/non-observable” and therefore more difficult to proxy-report. On the other hand, the mobility, self-care, and usual activities dimensions in EQ-5D are more “observable” and are therefore considered to be less subjective [10, 13]. This issue is particularly important in dementia, since people with more severe cognitive impairment may not be able to reliably self-report their HRQoL. Therefore, whilst patient self-report is usually considered preferable, this may not be feasible for all PwD.

In light of the challenges surrounding the use of self and proxy-rated HRQoL in dementia, there is the need to develop ways of overcoming these issues to ensure accurate and reliable analyses. It is important to retain the patient as the focus, therefore self-reports are considered default. However, to understand when it is better to use proxy-reports, dementia severity and dimension specific data should be explored. Therefore, there is a gap in the research to investigate the convergent validity of EQ-5D against core outcome measures in dementia for both self and proxy-reports. Although there are previous systematic reviews exploring the psychometric properties of EQ-5D [7–10], these do not specifically focus on convergent validity and hence the level of detail provided on convergent validity is limited. Convergent validity is an important property that, when explored in detail, may benefit researchers while choosing a HRQoL measure to use in trials, taking into consideration other instruments used in dementia studies. Therefore, this review will focus specifically on convergent validity of EQ-5D. This research adds to the existing psychometric literature of EQ-5D in dementia populations, and aids in addressing the question of how well EQ-5D captures dementia HRQoL in light of its widespread use. Therefore, a systematic literature review of the existing evidence was conducted, which to our knowledge is the first of its kind.

### Systematic review aim

The aim of this systematic review was to assess the convergent validity of the five EQ-5D dimensions (both 3L and 5L versions) with pre-defined core outcomes in dementia, taking into consideration the potential impacts of rater type (self vs. proxy EQ-5D reports).

## Methods

The systematic review adopted the methodology outlined by the Centre for Review and Dissemination (CRD) [15]. The Preferred Reporting of Items for Systematic Review and Meta-Analysis guidance (PRISMA) [16] were followed for reporting the results, and a narrative approach adopted for reporting the main analysis.

### Convergent validity

Convergent validity defines the strength of association between the measure of interest (in this case EQ-5D) and other measures via statistical significance of regression analyses or correlation coefficients. If the correlation with a measure capturing the same construct (kappa) is: > 0.4 it is considered as moderate and convergent validity is established (> 0.2: slight, > 0.6: good and > 0.8: very high) [17].

### Core dementia outcome measures

Although cognition is the hallmark feature of dementia, the original description and diagnosis also include functional and behavioural deficits, whereby dementia severity and progression is assessed by changes in one or more of these outcome areas [1]. To explore current practice for outcome collection in dementia clinical trials, various resources were searched and appraised via a previous review conducted as part of a wider research project, see Additional file 1 for further details. Table 1 below provides a summary of the key recurring outcome areas and measurement instruments identified as recommended, and considered as core for collection in dementia studies and trials.

### Literature searching

The literature search was conducted in three electronic databases (Medline, PsycINFO and CINAHL) by one author (HH) initially in April 2021 from database inception, and later re-ran and updated to September 2022. Search terms included those related to dementia, EQ-5D and core measure names. The full search strategy is provided in Additional file 2. Title and abstract screening were conducted by one author (HH) and verified independently by another author (AK) to check for discrepancies and establish study inclusion. Included studies were then screened in full text by one author (HH) against pre-defined inclusion and exclusion criteria to determine eligibility for the review. If at this point a text did not include extractable data, it was excluded from the review. Any discrepancies were discussed between the authors and resolved prior to data extraction.

### Inclusion/ exclusion criteria

Studies were considered eligible for inclusion within the review if they included individual level data from people with dementia of any type (as opposed to general ageing or mild cognitive impairment alone), they collected self and/or proxy reported EQ-5D-3L or EQ-5D-5L and they collected at least one of the predefined core dementia outcome measures (see Table 1). Studies reporting only EQ VAS were excluded as the purpose of the review is to focus on measures for use in economic evaluation, and EQ VAS cannot be used for this purpose. Studies collecting outcomes related to caregivers alone were excluded. To allow for diversity of the study types, all study designs were considered eligible for the review (e.g., observational studies and randomized controlled trials (RCT)) but were limited to those published in English (see Additional file 3). Protocol papers, feasibility studies, conference abstracts, grey literature and previous systematic reviews were excluded, but were chain searched for additional eligible references.

### Data extraction

Data on the study characteristics, settings, aspects of dementia (i.e., type and stage) and study objectives was synthesised using pre-defined extraction tables. Convergent validity of EQ-5D was captured against any additional reported core dementia outcome measures, as outlined in Table 1. Statistical data on relationships between the outcomes and EQ-5D index scores as well as with the EQ-5D dimensions were extracted. EQ VAS data were not extracted. A narrative synthesis was used to interpret the extracted data.

### Quality appraisal

The standardised GRADE assessment tool was adapted and used to assess the quality of the papers included in the review [18]. Although this method is less formal than using a pre-existing quality appraisal tool, it was deemed the most appropriate as the review allowed for the inclusion of all study types. The quality appraisal included nine items regarding the study’s population, sample, outcome assessment, analysis, and data resulting in a score of either high, medium, or low quality (see Additional file 4 for full details).

## Results

**Fig. 1:**
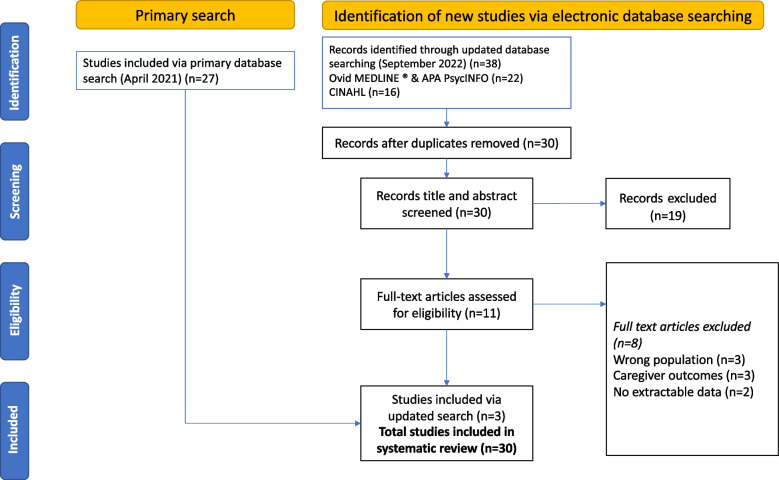
PRISMA flow diagram of literature searching hits

The outcome of the literature search and screening is shown in Fig. 1. The initial search strategy retrieved 282 records. Following the removal of duplicates there were 236 records remaining, for which the titles and abstracts were screened. After re-running and updating the search, 30 articles met the inclusion criteria for the review.

### Study characteristics

Table 2 provides a summary of the characteristics of the selected studies. Of the 30 studies included, they were predominantly from European countries (*n* = 15) [19–33] and the UK (*n* = 7) [34–40]. There were a total of 17 papers published since 2012 [20, 22, 23, 25–28, 31, 33, 35–43] (last ten years), and the most recent study included was from 2022 [40]. Less than a fifth of the studies used data from RCTs (*n* = 5) [20, 21, 29, 36, 37]. Alzheimer’s disease (AD) was the most included type of dementia, and it was the sole subtype evaluated in over a third of the studies (*n* = 11) [20, 25, 27, 28, 32, 39, 43–47] ; four studies considered AD in combination with dementia with Lewy Bodies (DLB) [21, 31], vascular dementia (VD) [34] or mixed [30]. Seven of the studies considered any type of dementia [19, 24, 33, 35, 37, 38, 40], and eight studies did not specify the type of dementia under investigation [22, 23, 26, 29, 36, 41, 42, 48].

**Table 2:** Summary characteristics of included studies

**Author, year**	**Country**	**Study design (of data origin)**	**n**	**Type dementia**	**Stage of dementia**	**Severity proportion (%)**	**Mean MMSE (SD)**	**Mean age (SD)**	**EQ-5D: 3L or 5L**	**Residence (% institutionalised)**	**Self or proxy QoL**	**Proxy type**	**Mode of administration**
Ankri, 2003 [19]	France	Non-interventional	142	Any	Mild-to-severe	24.3% mild; 47.1% moderate; 27.9% severe	12.8 (5.6)	82.9 (8.32)	3L	41	Both	FC & IC	I
Ashizawa, 2021 [43]	Japan	Prospective cohort study	287	AD	Mild-to-severe	NR	NR	86.1 (6.4)	5L	100	Proxy	FC & IC	SA
Bhattacharya, 2010 [20]	Denmark	RCT	321	AD	Mild	NR	24.0 (2.7)	76.2 (7.1)	3L	0	Both	IC	I
Bonfiglio, 2019 [41]	Japan	Cross-sectional	141	NR	Mild	NR	24.9 (2.6)	78.8 (6.3)	5L	0	Both	FC & IC	SA
Bostrom, 2007 [21]	Sweden	RCT	68	DLB or AD	Mild-to-severe	NR	16.9 (0–30)	77.8 (63–92)^c^	3L	28	Both	IC	I
Bryan, 2005 [34]	UK	Non-interventional	64	AD and VD	Very mild-to-moderate^a^	5% very mild; 56% mild, 39% moderate	18.0 (5.8)	76 (53–91)^c^	3L	0	Proxy	IC & C	SA
Castro-Monteiro, 2014 [22]	Spain	Multicentre longitudinal cohort	274	NR	Mild-to-severe^a^	18% mild; 26% moderate; 56% severe	NR	84.7 (6.51)	3L	100	Proxy	IC	I
Diaz-Redondo, 2014 [23]	Spain	Cross-sectional	525	NR	Mild-to-severe^a^	14% mild; 25% moderate; 62% severe	NR	85.6 (6.7)	3L	100	Proxy	IC	NR
**Author, year**	**Country**	**Study design (of data origin)**	**n**	**Type dementia**	**Stage of dementia**	**Severity proportion (%)**	**Mean MMSE (SD)**	**Mean age (SD)**	**EQ-5D: 3L or 5L**	**Residence (% institutionalised)**	**Self or proxy QoL**	**Proxy type**	
Easton, 2018 [42]	Australia	Cross-sectional	541	NR	Very mild-to-moderate	55% very mild; 42% mild; 4% moderate	NR	85.5 (8.5)	5L	100	Both	IC	I
Ersek, 2010 [24]	Hungary	Cross-sectional	88	Any	Very mild-to-severe	16% very mild; 27% mild; 25% moderate’; 16% severe	16.7 (7.2)	77.4 (9.2)	3L^b^	NR	NR	IC	NR
Farina, 2020 [35]	UK	Cohort	307	Any	Mild-to-severe	36% mild; 33% moderate; 32% severe	14.9 (9.1)	80.9 (8.4)	3L	21	Both	IC	SA
Garre-Olmo, 2017 [25]	Spain	Cross-sectional multicentre	343	AD	Mild-to-severe	32.1% mild; 36.7% moderate; 31.2% severe	14.2 (6.3)	78.9 (7.4)	3L	NR	Proxy	IC	I
Gonzalez-Velez, 2015 [26]	Spain	Multicentre longitudinal cohort	412	NR	Mild/moderate-to-severe^a^	44% mild/moderate; 56% severe	13.0 (8.5)	84.7 (6.5)	3L^b^	100	Proxy	FC & IC	SA
**Author, year**	**Country**	**Study design (of data origin)**	**n**	**Type dementia**	**Stage of dementia**	**Severity proportion (%)**	**Mean MMSE (SD)**	**Mean age (SD)**	**EQ-5D: 3L or 5L**	**Residence (% institutionalised)**	**Self or proxy QoL**	**Proxy type**	
Haaksma, 2018 [27]	Netherlands	Multicentre longitudinal prospective cohort	331	AD	Mild-to-moderate	65% very mild	21.9 (3.7)	74.9 (10.2)	3L	NR	Proxy	IC	I
Heßmann et al., 2016 [28]	Germany	Non-interventional	395	AD	Mild-to-severe	13% very mild; 33% mild; 27% moderate; 28% severe	18.8 (8.3)	79	3L	31	Both	IC	I
Karlawish, 2008 [1] [44]	USA	Non-interventional	110	AD	Very mild-to-moderate	32% very mild; 39% mild; 29% moderate	21.3 (4.3)	76.8 (2.7)	3L	0	Self	n/a	I
Karlawish, 2008 [2] [45]	USA	Non-interventional	110	AD	Very mild-to-moderate	28% very mild; 37% mild; 35% moderate	20.8 (4.4)	76.8 (2.7)	3L	0	Proxy	IC	I
King 2022 [40]	UK	Cohort study	243	Any	Mild-to-severe	~ 33% each for mild, moderate and severe	15.9 (9.1)	80.1 (8.6)	3L	18.7	Both	IC	I
Kunz, 2010 [29]	Germany	RCT	399	NR	Mild-to-moderate	65% mild; 35% moderate	18.6 (3.8)	80.2 (6.7)	3L	0	Both	IC	I
Kuo, 2010 [48]	Taiwan	Cost analysis	140	NR	Mild-to-severe	24% mild; 30% mild-moderate; 21% moderate; 25% severe	NR	79.7	3L^b^	36	Both	IC	I
**Author, year**	**Country**	**Study design (of data origin)**	**n**	**Type dementia**	**Stage of dementia**	**Severity proportion (%)**	**Mean MMSE (SD)**	**Mean age (SD)**	**EQ-5D: 3L or 5L**	**Residence (% institutionalised)**	**Self or proxy QoL**	**Proxy type**	
Martin, 2019 [36]	UK	RCT	1004	NR	Mild-to-severe	4% very mild; 23% mild; 39% moderate; 34% severe^a^	NR	85.5 (58–102.6)^c^	3L	100	Both	FC & IC	I
Michalowsky, 2021 [33]	Germany	Interventional study	77	Any	Mild-to-moderate	NR	18.6 (7.4)	80.2 (6.4)	Both	0	Both	FC & IC	I
Naglie, 2011 [1] [47]	Canada	Longitudinal cohort	370	AD	Very mild-to-moderate	71% very mild; 20% mild; 9% moderate	22.3 (4.3)	80.7 (7.8)	3L	0	Self	n/a	I
Naglie, 2011 [2] [46]	Canada	Longitudinal cohort	412	AD	Very mild-to-severe	64% very mild; 18% mild; 10% moderate; 8% severe	20.8 (6.2)	80.7 (7.9)	3L	0	Proxy	IC	I
Orgeta, 2015 [37]	UK	RCT	478	Any	Mild-to-moderate^a^	75% mild; 25% moderate	NR	75.5 (7.3)	3L	0	Both	IC	I
Schiffczyk, 2010 [30]	Germany	Prospective cohort	137	AD or mixed	Mild-to-severe	37% mild; 48% moderate; 15% severe	16.9 (6.4)	69.9 (7.6)	3L	0	Both	IC	I
**Author, year**	**Country**	**Study design (of data origin)**	**n**	**Type dementia**	**Stage of dementia**	**Severity proportion (%)**	**Mean MMSE (SD)**	**Mean age (SD)**	**EQ-5D: 3L or 5L**	**Residence (% institutionalised)**	**Self or proxy QoL**	**Proxy type**	
Sheehan, 2012 [38]	UK	Non-interventional	112	Any	Mild-to-severe^a^	2% very mild; 13% mild; 59% moderate; 27% severe	NR	85 (66–99)^c^	3L	25	Both	IC	I
Trigg, 2015 [39]	UK	Multicentre cohort	145	AD	Very mild-to-severe	NR	15.0 (7.0)	77.8 (9.2)	3L	NR	Both	Unclear	I
van de Beek, 2019 [31]	Netherlands	Multicentre cohort	138	AD and DLB	MCI or dementia	NR	25 [22-27]	69.7 (5.9)	3L	NR	Self	n/a	NR
Vogel, 2006 [32]	Denmark	Prospective cohort	48	AD	Very mild	NR	24.9 (2.3)	77.0 (5.8)	3L	NR	Both	IC	PwD – I; Proxy – SA

*AD* Alzheimer’s disease, *C* Clinician, *DLB* Dementia with Lewy bodies, *FC* Formal caregiver, *IC* Informal caregiver, *QoL* Quality of life, *RCT* Randomised controlled trial, *UK* United Kingdom, *USA* United states of America, *VD* Vascular dementia, *3L* Three-level, *5L* Five-level, *NR* Not reported, *n/a* Not applicable, *MCI* Mild cognitive impairment, *I* Interviewer, *SA* Self-administered

^a^Disease severity defined by CDR (versus MMSE in all other studies)

^b^EQ-5D version not reported, however due to year of the studies – EQ-5D-3L has been assumed

^c^Study reported range (not standard deviation)

The studies included samples across all stages of dementia, from very mild to severe, which was typically characterised by MMSE scores. Most of the studies defined dementia severity via MMSE scores, however some studies did not collect MMSE, thereby using an alternative outcome, i.e., CDR [22, 23, 26, 34, 37, 38]. The lowest mean MMSE reported was 12.8 [19], and the highest was 24.9 [32, 41]. The study sample sizes ranged from 48 [32] – 1004 [36] participants. EQ-5D-3L was most commonly collected (*n* = 27), and only four studies collected EQ-5D-5L [33, 41–43] (one study considered both EQ-5D-3L and EQ-5D-5L [33]). There was a mix of study settings; eleven studies focused solely on community dwelling PwD [20, 29, 30, 33, 34, 37, 41, 44–47], while six studies collected data from institutionalised residents alone [22, 23, 26, 36, 42, 43]. Quality of life data were collected entirely via a proxy (*n* = 9) [22, 23, 25–27, 34, 43, 45, 46] or self-report (*n *= 3) [31, 44, 47] in some studies, however over half of the studies used both proxy and self-report (*n* = 17) [19–21, 28–30, 32, 33, 35–42, 44, 45]. Where self-report was exclusively used, the studies explored people with mild dementia. Of the seventeen studies that included people with severe dementia within their sample, ten studies used both self and proxy reports [19, 21, 28, 30, 35, 36, 38–40, 48], six studies used proxy-reports alone [22, 23, 25, 26, 43, 46] and one study did not report the rater type [24]. The proxy-type was most commonly an informal caregiver (i.e., family member, friend, or neighbour), however four studies also included formal caregivers [19, 26, 36, 41] and one study additionally used clinicians to proxy report [34]. EQ-5D was mainly administered was via an interview (*n* = 21) [19–22, 25, 27–30, 32, 33, 36–40, 42, 44–48], three studies did not report this detail [23, 24, 31], and one study used interviews for PwD and self-administration for proxies [32]. Of the four studies that solely used PwD self-reported EQ-5D, two collected this via interview [44, 46], one used self-administration booklets [43] and one did not report this information [31].

## Core dementia outcome measures

Table 3 lists the measures that were used to measure the core dementia outcomes in the studies. Details of the measures are provided in Additional file 5. In total there were 12 distinct measures: Two cognition measures (MMSE and ADAS-Cog), six measures of function (Katz ADL, ADCS-ADL, Barthel index, Lawton scale, DAD and BADLS) and four behaviour/mood measures (NPI, CSDD, GDS and CMAI). Where it was reported, the measures were completed either by proxy, researcher observation, or a combination of information such as self or proxy information and recent care records (administration details are provided in Additional file 5). The most predominant measure was the MMSE (*n* = 25), followed by the NPI (*n* = 12).

**Table 3:** Core dementia outcome measures

	**Cognition**		**Function**						**Behaviour/mood**			
**Author**	**MMSE**	**ADAS-Cog**	**Katz index**	**ADCS-ADL**	**Barthel index**	**Lawton scale**	**DAD**	**BADLS**	**NPI**	**CSDD**	**GDS**	**CMAI**
Ankri, 2003 [19]	✓		✓									
Ashizawa, 2021 [43]	✓				✓							
Bhattacharya, 2010 [20]	✓			✓					✓	✓		
Bonfiglio, 2019 [41]	✓				✓	✓					✓	
Bostrom, 2007 [21]		✓					✓		✓			
Bryan, 2005 [34]	✓							✓	✓			
Castro-Monteiro, 2014 [22]	✓				✓					✓		
Diaz-Redondo, 2014 [23]	✓				✓					✓		
Easton, 2018 [42]					✓				✓			
Ersek, 2010 [24]	✓											
Farina, 2020 [35]	✓								✓			
Garre-Olmo, 2017 [25]	✓						✓		✓			
Gonzalez-Velez, 2015 [26]	✓				✓					✓		
Haaksma, 2018 [27]	✓						✓		✓			
Heßmann, 2018 [28]	✓			✓					✓			
Karlawish, 2008 [1] [44]	✓					✓					✓	
**Author**	**MMSE**	**ADAS-Cog**	**Katz index**	**ADCS-ADL**	**Barthel index**	**Lawton scale**	**DAD**	**BADLS**	**NPI**	**CSDD**	**GDS**	**CMAI**
Karlawish, 2008 [2] [45]	✓					✓					✓	
King, 2022 [40]	✓							✓				
Kunz, 2010 [29]	✓				✓							
Kuo, 2010 [48]	✓				✓							
Martin, 2019 [36]												✓
Michalowsky, 2021 [33]	✓										✓	
Naglie, 2011 [1] [47]	✓	✓					✓		✓		✓	
Naglie, 2011 [2] [46]	✓	✓					✓		✓			
Orgeta, 2015 [37]								✓		✓		
Schiffczyk, 2010 [30]	✓	✓									✓	
Sheehan, 2012 [38]						✓					✓	
Trigg, 2015 [39]	✓						✓		✓			
van de Beek, 2019 [31]	✓						✓		✓		✓	
Vogel, 2006 [32]	✓										✓	
Number of studies	25	4	1	2	8	4	7	3	12	5	9	1

The MMSE and ADAS-Cog measures commonly capture cognitive impairment, however the latter is administered via direct observation, resulting in a longer administration duration. There two types of daily activities – basic activities of daily living (BADLs) and instrumental-ADLs. Instrumental ADLs are not necessary for fundamental functioning, they are generally more complex activities that allow a person to live independently, e.g., managing one’s own finances. Basic ADLs are fundamental skills, typically related to basic physical needs, e.g., toileting and eating [49]. Of the six function measures, two captured basic ADLs (BADL) alone (Katz ADL and Barthel index), one captured instrumental ADLs (IADL) alone (Lawton scale) and the remaining three included both BADL and IADL items (ADCS-ADL, DAD and BADLS). Of the behavioural measures, two measured depression (CSDD and GDS), while the others captured agitation (CMAI) and general neuropsychiatric symptoms (NPI).

### EQ-5D convergent validity with cognition

It was hypothesised that cognition would have a positive correlation with EQ-5D whereby greater cognitive impairment would be associated with lower EQ-5D index scores (lower MMSE scores indicate greater cognitive impairment). Additional file 6 provides complete details of the empirical relationship between cognition and EQ-5D. In total, eighteen studies assessed the convergent validity between EQ-5D index scores and the cognitive measures (MMSE, *n* = 17; ADAS-Cog, *n* = 2; one study collected both measures [41]). Three studies reported a different relationship between cognition and EQ-5D by rater type [28, 35, 41].

**Fig. 2:**
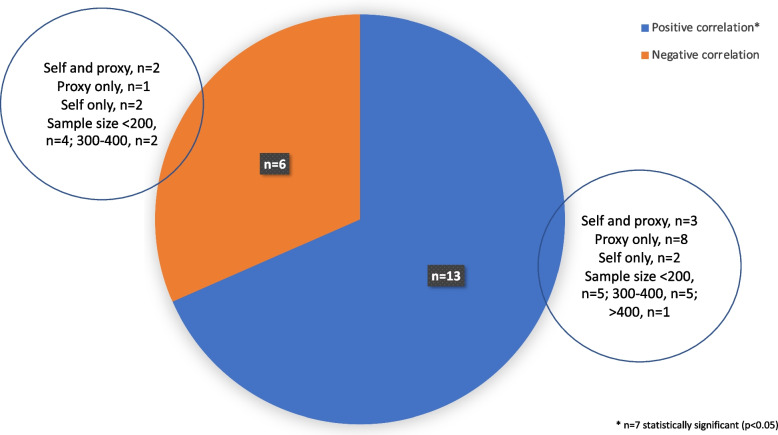
Direction of convergent validity between EQ-5D and cognition. *Total number of studies is *n* = 19 as 2 studies report convergent validity twice

Within only seven distinct studies a statistically significant relationship (*p* < 0.05) between cognition and EQ-5D was reported, all of which were *positive correlations* [28, 30, 35, 40, 41, 43, 46], and three of these seven studies had a sample size of greater than 300 [28, 35, 46]. Of the studies that reported statistically significant findings, the rater type was predominantly an informal caregiver (5/7 studies), and were studies that had included participants spanning the entire dementia severity range (mild-to-severe). The one study that reported a statistically significant association between self-reported EQ-5D and cognition was within a mild-stage study sample [41]. Figure 2 shows the proportion of studies that demonstrated a relationship between cognition and EQ-5D in both directions (see Additional file 6 for more details).

### EQ-5D convergent validity with function

For the convergent validity between EQ-5D and the measures of function, a positive correlation was hypothesised whereby greater functional independence would be associated with higher EQ-5D index scores (see Additional file 5 for details of function instrument scoring). Twenty distinct studies provided empirical evidence of the convergent validity between EQ-5D and the measure of function within the study (ADCS-ADL, *n* = 2; BADLS, *n* = 2; Barthel index, *n* = 7; DAD, *n* = 6; Lawton scale, *n* = 4; one study collected both Barthel index and Lawton scale [41]).

**Fig. 3:**
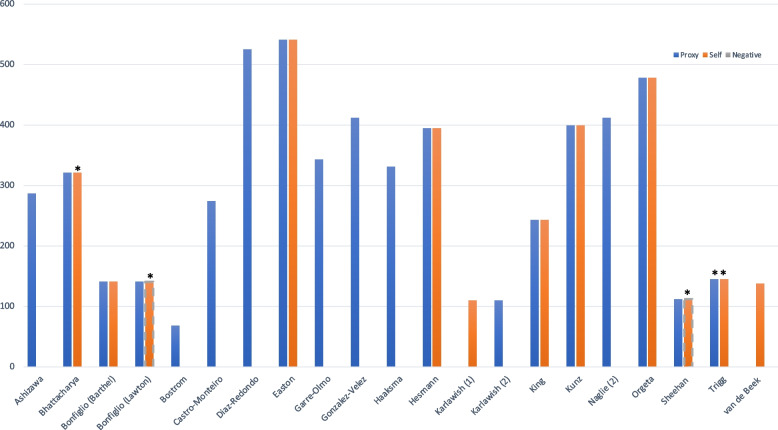
Characteristics of studies reporting convergent validity between EQ-5D and function*.* *Indicates non-significant correlation (*p* > 0.05). Y axis = sample size

Two studies reported a difference in relationship between function and EQ-5D by rater type, whereby the Lawton index showed a positive and significant correlation with proxy EQ-5D and a negative and non-significant correlation with self-rated EQ-5D [38, 41]. These two studies were the only reports of a negative association; both of these studies had a mainly mild-stage sample size of < 200 participants. The remaining studies all reported a positive correlation between function and EQ-5D, of which the majority (15/16 studies) were statistically significant. Two studies explored function as a dependent variable within regression analyses, both of which found it to be a significant (*p* < 0.01) determinant of proxy reported EQ-5D [40, 43], but not self-reported EQ-5D [40]. One study had reported a positive correlation between ADCS-ADL and EQ-5D for both rater types, but was only statistically significant for proxy-report [20], and was again within a mild-stage study sample [20]. Figure 3 shows the characteristics of the studies that reported convergent validity evidence between function and EQ-5D (Additional file 7 provides complete details).

### EQ-5D convergent validity with behaviour/mood

For the behaviour/mood measures, higher scores indicate greater severity (see Additional file 5). Therefore, it was hypothesised that the measures would have negative correlations with EQ-5D, whereby more behavioural disturbance is associated with lower EQ-5D index scores. Seventeen distinct studies reported empirical evidence of the convergent validity between EQ-5D and the measure of behaviour/mood, four studies collected multiple measures [20, 28, 46, 47] (CSDD, *n* = 2; CMAI, *n* = 1; GDS, *n* = 8; NPI, *n* = 10).

Only one study captured agitation (via CMAI), reporting a negative correlation with EQ-5D which was only statistically significant for proxy-report [36].

Ten studies measured depression (via CSDD and GDS). All ten studies reported a negative correlation between the measure of depression and EQ-5D, whereby statistically significant results were found with self-rated EQ-5D only *n *= 4 [31, 41, 44, 47]; proxy-EQ-5D only, *n* = 2 [37, 46] and both rater types, *n* = 2 [20, 28]. One study did not report statistical significance, but rather strength of correlation coefficients – indicating moderate convergent validity between EQ-5D index scores and GDS [33].

**Fig. 4:**
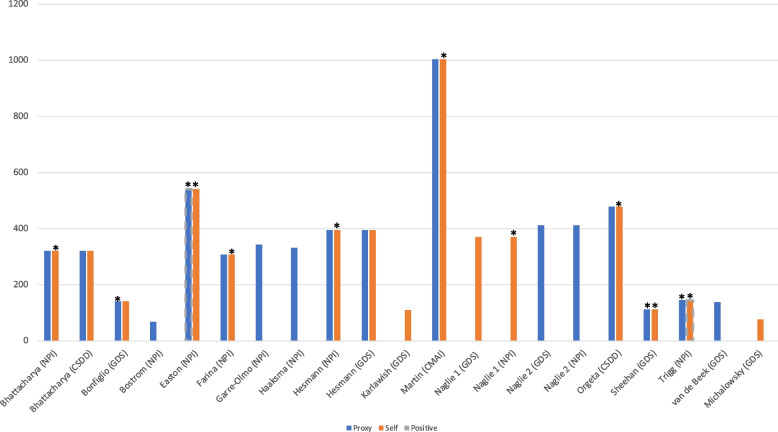
Characteristics of studies reporting convergent validity between EQ-5D and behaviour*.* *Indicates non-significant correlation (*p* > 0.05). Y axis = sample size

The NPI captures 12 broad neuropsychiatric symptoms and was administered in ten of the reviewed studies. Two of these studies reported a difference in relationship between NPI and EQ-5D by rater type; one study found a negative correlation with self-rated EQ-5D, but a positive correlation with proxy-EQ-5D [42], while the other study found the inverse [39]. However, neither of these findings were statistically significant. The remaining studies all reported negative correlations, whereby statistical significance was found only with proxy-EQ-5D [20, 21, 25, 27, 28, 35, 46]. Figure 4 shows the characteristics of the studies that reported convergent validity evidence between the behaviour/mood measure and EQ-5D (Additional file 8 provides complete details).

### Convergent validity evidence by EQ-5D dimension

A total of seven distinct studies reported empirical evidence of convergent validity of the pre-defined core dementia outcome measures with EQ-5D dimensions – summarised in Table 4. Cognition (via MMSE) was associated with self-rated anxiety/depression [19], and people with more cognitive impairment self-reported fewer problems across all EQ-5D dimensions [42].

**Table 4:** Empirical evidence of relationships between outcome measures and EQ-5D dimensions

**Broader domain**	**Empirical evidence of association with EQ-5D dimensions**	**Proxy**	**Self**	**Reference**
**Cognition**	Positive association between MMSE and anxiety/ depression **(*F* = 6.86, *p* = .001)**	◦◦	✓	Anrki et al. [19]
	People with severe cognitive impairment (MMSE 0–9) reported considerably fewer problems in all EQ-5D dimensions – compared with the less cognitively impaired. People with MMSE 20–30 reported the most problems with pain/discomfort. People with MMSE 10–19 had the most problems with usual activities		✓	Heßmann et al. [28]
**ADL**	Positive association between Katz indicator and mobility **(*F* = 16.4, *p* < .0001)**		✓	Ankri et al. [19]
	Positive association between Katz indicator and self-care **(*F* = 6.3, *p* < .0001)**		✓	Ankri et al. [19]
	Positive association between Katz indicator and usual activities **(*F* = 6.8, *p* < .002)**		✓	Ankri et al. [19]
	Positive association between Katz indicator and pain/discomfort **(*F* = 6.8, *p* < .002)**		✓	Anrki et al. [19]
	Boxplots indicate positive association between BADLS and mobility. Observed associations: all carers 0.44; clinician 0.59; live-in carers 0.38; **(*p* < 0.01)**	✓		Bryan et al. [34]
	Boxplots indicate positive association BADLS and self-care. Observed associations: all carers 0.57; clinician 0.62; live-in carers 0.65; (***p* < 0.01)**	✓		Bryan et al. [34]
	Boxplots indicate positive association BADLS and usual activities. Observed associations: all carers 0.62; clinician 0.50; live-in carers 0.75; (***p* < 0.01)**	✓		Bryan et al. [34]
	Reporting problems in all EQ-5D dimension (minus anxiety/depression) was significantly associated with worse scores on the Barthel index **(*p* < 0.01)**	✓		Diaz-Redondo et al. [23]
	Negative association between Barthel index score and self-reported mobility **(-0.499),** self-care **(-.0609)** and usual activities **(-0.374) = *p* < 0.01**		✓	Easton et al. [42]
	Negative association between Barthel index score and proxy reported mobility **(-0.555**), self-care (-**0.627),** usual activities **(-0.577) = *p* < 0.01**	✓		Easton et al. [42]
**Behaviour/mood**	NPI summary score and EQ-5D anxiety/depression show positive association. Observed associations seen between clinician proxy rating = **0.46, *p* < 0.01.** No association for other proxy types	✓		Bryan et al. [34]
	Reporting problems in EQ-5D anxiety/depression dimension was significantly associated with Cornell scale score **(*p* < 0.01)**	✓		Diaz-Redondo et al. [23]
	NPI negatively association with mobility **(-0.177)** and anxiety/depression **(0.160) = *p *< 0.01**	✓		Easton et al. [42]
	GDS demonstrated moderate convergent validity via strength of correlation coefficients with the following EQ-5D dimensions: mobility (3L), **0.310**; self-care (3L), **0.446**; usual activities (3L), **0.414;** anxiety/depression (5L), **0.317;** poor correlation with pain/discomfort – ***p* values not reported**		✓	Michalowsky et al. [33]

Function via the Katz index was associated with self-rated mobility, self-care, usual activities, and pain/discomfort; no relation was found with anxiety/depression [19]. Function via BADLS was correlated with proxy rated mobility, self-care and usual activities. Stronger correlations were observed for informal carer reports of self-care and usual activities, while the clinician rated mobility correlation was stronger [34]. Function via Barthel index was significantly correlated with self and proxy mobility, self-care and usual activities [42], and was associated with reporting problems in all EQ-5D dimensions minus anxiety/depression [23].

Depression (via CSDD) was associated with reporting problems in anxiety/depression [23]; and depression (via GDS) showed evidence of moderate convergent validity with mobility, self-care, usual activities and anxiety/depression [33]. NPI summary scores were associated with proxy rated anxiety/depression [34, 42] and mobility [42].

### Inter-rater agreement

To further understand the potential impacts of rater-type upon EQ-5D assessment, information related to the inter-rater agreement was extracted and is summarised in Table 5.

**Table 5:** Evidence of inter-rater agreement

	**Inter-rater agreement (PwD and proxy)**	
**EQ-5D dimension**	Evidence	Source
**Mobility**	Mobility was the only dimension to produce an acceptable level of agreement (kappa = 0.53 formal, 0.44 informal proxy)	Ankri et al. [19]
	Mobility dimension produced the best agreement on the dimensions (kappa coefficient indicate moderate agreement)	Orgeta et al. [37]
	Proxies rated more problems in the mobility dimension (than PwD had self-rated)	Vogel et al. [32]
**Self-care**	PwD self-rated self-care significantly more optimistically than proxies (1.0 ± 0.2 vs 1.1 ± 0.4, *P* = 0.031	Bonfiglio et al. [41]
	Self-care was the only EQ-5D dimension to showed a significant correlation between self and proxy report (*r *= 0.51, *p* < 0.01)	Vogel et al. [32]
**Usual activities**	Agreement (across proxy type) was lowest with PwD for usual activities dimension	Orgeta et al. [37]
	The difference between the kappa-coefficients in the subgroups of mild vs moderate PwD was statistically significant (*p* > 0.05)	Kunz et al. [29]
	PwD self-rated usual activities significantly more optimistically than proxies (1.1 ± 0.4 vs 1.6 ± 0.8, *P* = 0.000)	Bonfiglio et al. [41]
	Proxies rated more problems in the usual activities dimension (than PwD had self-rated)	Vogel et al. [32]
	PwD self-rated pain/discomfort significantly more optimistically than proxies (1.5 ± 0.7 vs 1.7 ± 0.9, *P* = 0.015)	Bonfiglio et al. [41]
	Proxies rated more problems in the pain/discomfort dimension (than PwD had self-rated)	Vogel et al. [32]
**Anxiety/depression**	PwD self-rated anxiety/depression significantly more optimistically than proxies (1.1 ± 0.5 vs 1.3 ± 0.5, *P* = 0.008)	Bonfiglio et al. [41]
	Anxiety/depression was the only dimension that PwD self-rated more problems than proxies	Vogel et al. [32]
**EQ-5D index score**	Intraclass correlation coefficient for EQ-5D total scores on PwD and proxy responses reflected average concordance – informal: ICC = 0.41, *p* < 0.001), formal: ICC = 0.42, *p* < 0.001)	Ankri et al. [19]
	Proxy EQ-5D ratings were significantly worse, with a mean difference of 0.1 in total score	Kunz et al. [29]
	Relationships between EQ-5D scores and clinical variables (CSDD, NPI, ADCS-ADL) were stronger for proxy assessments	Bhatttacharya et al. [20]
	Proxy EQ-5D index scores were significantly lower than self-report (0.8 ± 0.1 vs 0.9 ± 0.1, *P* = 0.000)	Bonfiglio et al. [41]
	MMSE and NPI scores were significantly associated with EQ-5D proxy (*p* = 0.00), but not EQ-5D self report (*p* = 0.63	Farina et al. [35]
	EQ-5D index scores were significantly different based on the rater: 0.67 (± 0.33) for self-report and 0.45 (± 0.36) for proxy, *p* < 0.001	Heßmann et al. [28]
	Self-completed EQ-5D was poor at reflecting clinically important differences and changes in clinical measures, vs EQ-5D proxy which did capture these changes	Martin et al. [36]
	EQ-5D proxy index scores were significantly lower than self-scores	Orgeta et al. [37]
	Self-rated EQ-5D scores were significantly higher than proxy EQ-5D (patient mean EQ5D score 0.71, 95% CI 0.64–0.77, proxies mean EQ5D score 0.30, 95% CI 0.22–0.38), mean difference 0.40 (95% CI 0.32–0.48, p 5 0.001)	Sheehan et al. [38]

Nine studies, representing samples across the entire dementia-severity range, commented on the inter-rater agreement between self and proxy rated EQ-5D index scores [19, 28, 29, 32, 35–38, 41]. Proxy-EQ-5D index scores were found to be significantly lower than self-report [28, 29, 37, 38, 41] and had stronger correlations with clinical variables [20, 35, 36].

Of the EQ-5D dimensions, it was reported in two distinct studies that the mobility dimension had the strongest inter-rater agreement, produced at an acceptable level (kappa > 0.4) (versus the other dimensions) [19, 37]. One of the studies reported that agreement between formal and informal proxies was also highest for mobility (kappa = 0.61), and all other dimensions remained below the usually accepted level [19].

Agreement between reports of the usual activities dimension was the lowest, with self-report reflecting more optimistic reports [29, 32, 37, 41]. Agreement in the pain/discomfort dimension was low, whereby proxies rated more problems than PwD themselves [32, 37, 41]. Evidence of agreement in the anxiety/depression dimension was mixed; one study found that PwD self-rated this dimension more optimistically than proxies [41], while another study reported that this was the only dimension that PwD had self-rated more problems (than proxies) [32].

### Quality appraisal

Of the 30 papers included within the review, 18 were of high quality, and the remaining 12 were considered to be of medium quality (see Additional file 4 for full quality appraisal).

## Discussion

There is a growing recognition of the wide use and acceptability of EQ-5D within dementia populations, thereby capturing generic HRQoL that can be converted to utilities for use in cost-effectiveness analyses. An important factor in exploring the use of such a measure is its psychometric properties. This targeted literature review identified 30 studies which contained empirical evidence related to the convergent validity of EQ-5D with at least one pre-defined core measure of: cognition (*n* = 18), function (*n* = 20), or behaviour/mood (*n* = 17), the main clinical outcomes in studies of people with dementia. The findings indicate that EQ-5D convergent validity with clinical measures of function, behaviour/mood and cognition were in the expected direction, whereby increased clinical impairment was associated with lower EQ-5D index score. There is clear evidence on the absence of inter-rater agreement between self and proxy report. Evidence at the dimension-level was limited, however there were some data to support convergent validity between specific EQ-5D dimensions and clinical outcomes, and differences in inter-rater agreement by dimension type.

It was hypothesised that as cognition deteriorates, EQ-5D would also deteriorate – indicating a positive correlation. If a switch occurs from self to proxy report when a PwD is no longer able to accurately self-report, this relationship would still be sustained. Only seven studies reported evidence of statistically significant associations with cognition, and they were all with a positive correlation, therefore agreeing with the a *priori* hypothesis. As observed and expected, proxy-reports showed stronger associations with the clinical measure. Where a negative but non-significant correlation with self-rated EQ-5D (within the same dyad) was reported [35, 41]; this finding could indicate that self-rated EQ-5D was collected until a certain stage of dementia severity before a switch to proxy-EQ-5D was initiated. However, papers did not tend to report this substitution. A negative relationship between self-rated EQ-5D and cognition would only be anticipated at the more severe stages of dementia progression if self-report were to still be used (where the PwD’s self-awareness has deteriorated) [2, 35]. In addition, a relationship between cognition and EQ-5D dimension: anxiety/depression was observed – whereby reporting more problems in this dimension corresponded with greater MMSE scores (less cognitive impairment) [19]. These findings potentially highlight the greater self-awareness at earlier cognitive stages, whereby the person can recognise their own deterioration, as well as newly identifying with the label of the dementia diagnosis (thereby inducing anxiety/depression).

The evidence on the convergent validity between EQ-5D and function was more robust. Fifteen studies reported a statistically significant positive association between the outcomes, whereby greater functional impairment was correlated with lower EQ-5D as hypothesised. This finding is echoed within the wider literature whereby using ADL as a marker of disease progression within economic models has been suggested, due to its importance within dementia disease experience and its alignment with HRQoL [50]. Only two studies reported a negative association with self-rated EQ-5D, however both findings were from mild-stage study samples, where it would be expected that functional impairment would be relatively low. Overall, the studies that reported non-significant results were mainly (75%) from sample sizes of < 200, further highlighting the challenges associated with considering smaller studies for psychometric appraisals. As outlined earlier, there are two fundamental types of ADL: BADLs and IADLs. Where there were mixed reports such that proxy-EQ-5D correlated with the measure of function, but self-rated EQ-5D did not, the instruments were measures of IADL (ADCS-ADL [20, 28] and Lawton Scale [38, 41]). As IADLs are daily tasks that are not necessary for functional living, e.g., handling finances, it may be that PwD do not recognise their impairments in conducting these activities as they are now being performed by a proxy, and that the proxy is more aware of these impairments and their impact. All the studies that measured BADLs showed evidence of convergent validity with EQ-5D. In dementia progression, IADL impairment is experienced sooner and BADLs are not impacted until the more severe stages of disease [51, 52]. Therefore, the relationship between EQ-5D with BADLs is not unanticipated, with EQ-5D being a measure of health status, it has been found to be more responsive to changes in severe stages of disease [53].

It was hypothesised that lower EQ-5D scores would be associated with greater behavioural/mood disturbances, as demonstrated by a negative correlation. The included measures captured different aspects of behaviour/mood. The only study where agitation (via CMAI) was measured reported a statistically significant relationship with EQ-5D-proxy [36]. Similarly, the evidence of convergent validity between neuropsychiatric disturbance (via NPI) and EQ-5D index scores was only statistically significant for proxy-report. Whereas evidence of EQ-5D convergent validity with measures of depression was observed to a greater extent with self-report. As depression is a more personal experience, and the NPI additionally captures broader neuropsychiatric symptoms, this pattern of association is predictable. However, this finding should be interpreted with caution as it is important to consider the impact of the administration of these clinical measures. The measures of depression are self-rated by the PwD themselves, while the broader clinical measures such as the NPI are informant-based, thereby completed by a proxy. Therefore, the relationships observed may be impacted by *who* is completing both measures, as opposed to the content of the measures themselves.

A key objective of this review was to explore the evidence surrounding convergent validity of EQ-5D dimensions with the core dementia outcome measures. The findings show that while the “observable” dimensions mobility, self-care and usual activities were associated with functional measures, the “non-observable” dimension anxiety/depression was associated with the measures of cognition and behaviour/mood. Although these findings indicate relationships between the clinical measures and the appropriate corresponding EQ-5D dimensions (thus demonstrating convergent validity of EQ-5D at the dimension-level), they should be interpreted with caution. Firstly, one paper reported that GDS had moderate convergent validity with all EQ-5D dimensions (minus pain/discomfort) [33]. Secondly, as highlighted by Karlawish et al., 41% of EQ-5D scores were at 1.0 (perfect health), and people did not self-report impairment in dimensions such as usual activities, where one would typically expect to see disability in this population [44]. Lastly, the number of papers reporting evidence at the EQ-5D dimension level was low (*n* = 7) and as indicated by Michalowsky et al., there are additional factors to consider when observing dimensions on both EQ-5D-3L and EQ-5D-5L [33].

Evidence assessing inter-rater agreement between self and proxy reports of EQ-5D index scores and where possible, EQ-5D dimensions mirrors the existing evidence – inter-rater agreement was generally poor, particularly for EQ-5D index scores. People with dementia self-rated fewer problems than their proxies. This finding is established within the literature [10, 14, 54] and is theorised to be the result of various factors such as response-shift [55, 56] and/or proxy burden resulting in “projection bias” [54, 57, 58].

When analysing inter-rater agreement at the dimension-level the findings were mixed. For the “non-observable” dimensions of pain/discomfort and anxiety/depression, agreement was low. This was also the case for the self-care dimension. The “observable” dimension of mobility was reported to have the strongest inter-rater agreement. Bonfiglio et al. commented on this phenomenon, highlighting the difficulty in establishing agreement between people with dementia and proxies for non-observable factors such as pain/discomfort and anxiety/depression [41]. Overall, proxy-assessments tend to show a higher degree of association with the clinical measures, an observation that has been recurrently noted in the wider literature [13, 14, 59, 60] and may be influenced by disease severity. Garre-Olmo et al. reported that strength of the relationship between function (via DAD) and EQ-5D increased with increasing disease severity [25]. However, exploring the impact of disease severity was beyond the scope of this review.

### Strengths and limitations

A key strength of this review is that it addresses a research area that has not been fully explored, and is to our knowledge, the first review of its kind to specifically synthesise the relevant evidence of EQ-5D convergent validity with dementia outcomes. However, the search strategy was only applied to limited electronic databases and therefore there may have been relevant studies that were not identified for this review (additional chain-searching of references was performed in an aim to minimise this). Only published journal articles in English language were included, resulting in a total of only 30 papers, and 25 papers were excluded solely because of the absence of extractable data. This highlights the lack of research focusing exclusively on psychometric properties, as this was a specified objective of only *n* = 16 papers, with the review evidence originating mainly from trials and observational studies. However, despite the lack of studies with a specific psychometric focus, over half of the included studies were deemed as high quality for evaluating convergent validity. In addition, as the core dementia outcome measures considered within this review were pre-determined (as described in Additional file 1), it is possible that not all measures were covered in the evaluation. However, to mitigate against this potential bias, a methodological approach was adopted in selecting the core outcome measures for consideration.

The inclusion criteria were broad, a range of study types were included, and no date restriction was applied to the online databases. Another strength was the exploration of inter-rater reliability and extracting this information at the EQ-5D dimension-level where available. Lastly, there are several factors that were not explored as they were beyond the scope of this review. The characteristics of the proxy (i.e., sociodemographic factors) and pragmatic aspects i.e., instrument administration method were not explored, which could potentially impact the findings. A previously published study reported that where carers themselves were in pain [61], they reported more problems with pain in the PwD, inferring projection bias. Additionally, one of the studies commented on the difference in validity by proxy type, reporting that the data provided by clinician-proxies, when compared to that of informal caregivers, had higher construct validity for the more “observable” dimensions of EQ-5D (i.e., mobility and self-care). On the other hand, data from informal caregivers had higher construct validity for the less observable dimensions (i.e., anxiety/depression).

## Conclusion

This systematic literature review concludes that there is published evidence to indicate convergent validity between EQ-5D and the core dementia outcome measures of function, behaviour/mood and cognition in the expected directions. Additionally, at the dimension-level, EQ-5D dimensions show associations with specific clinical measures, whereby the degree of association differed by rater-type (self vs. proxy raters). Overall inter-rater agreement was poor, particularly for the “non-observable” dimensions of EQ-5D (i.e., pain/discomfort, anxiety/depression). It should, however, be noted that these conclusions are based on very limited published data, and this review highlights the lack of studies investigating the psychometric properties of measures for use in dementia populations.

Measuring HRQoL in dementia populations is a complex issue, particularly when considering the challenges with both self and proxy reporting. This review revealed that, despite the limited evidence pool, EQ-5D exhibits convergent validity with other dementia outcomes, and that for “observable” dimensions (i.e., mobility, self-care, usual activities), the associations are stronger when using proxy-EQ-5D. There is currently no guidance on which report to use in evaluations and how to combine scores. Future empirical studies could investigate the severity range for which EQ-5D self-report could be reliably used, and the severity stage at which proxy-report become more appropriate.

## Supplementary Information

**Additional file 1.** Core outcome measures in dementia studies and trials.

**Additional file 2.** Complete search strategy.

**Additional file 3. **

**Additional file 4.** Quality assessment of included papers adapted from the GRADE assessment tool.

**Additional file 5. **Summary of core outcome measures.

**Additional file 6. **Evidence of EQ-5D convergent validity with cognitive measures.

**Additional file 7. **Evidence of EQ-5D convergent validity with function measures.

**Additional file 8. **Evidence of EQ-5D convergent validity with behaviour measures.

## Data Availability

Not applicable

## Abbreviations

AD: Alzheimer’s disease
ADL: Activities of daily living
ADAS-Cog: The Alzheimer's Disease Assessment Scale-Cognitive Subscale
ADCS-ADL: Alzheimer’s Disease Cooperative Study Activities of Daily Living Scale
BADL: Basic activity of daily living
BADLS: Bristol Activities of Daily Living Scale
CINAHL: Cumulative Index to Nursing and Allied Health Literature
CMAI: Cohen-Mansfield Agitation Inventory
CSDD: Cornell Scale for Depression in Dementia
DAD: Disability Assessment for Dementia
DLB: Dementia with lewy bodies
GDS: Geriatric Depression Scale
CRD: Centre for Review and Dissemination
HRQoL: Health-related quality of life
IADL: Instrumental activity of daily living
MMSE: Mini-Mental State Examination
MoCA: Montreal Cognitive Assessment
NICE: National Institute of Health and Care Excellence
NPI: Neuropsychiatric Inventory
PRISMA: The Preferred Reporting of Items for Systematic Review and Meta-Analysis guidance
PwD: Person with dementia
QoL: Quality of life
QoL-AD: Quality of Life in Alzheimer's disease scale
QUALID: Quality of Life in Late-Stage Dementia Scale
QUALIDEM: Quality of Life in Late-Stage Dementia Scale proxy
RCT: Randomised controlled trial
SIB: Severe Impairment Battery
VD: Vascular dementia
