# Supplementary material for: Convergent validity of EQ-5D with core outcomes in dementia: a systematic review
Source: Health Qual Life Outcomes. 2022 Nov 19;20:152. doi: 10.1186/s12955-022-02062-1 (PMC9675120; doi:10.1186/s12955-022-02062-1)
Supplement: Supplementary file 1 — Additional file 1. Core outcome measures in dementia studies and trials. [file 12955_2022_2062_MOESM1_ESM.docx]

**Additional File 1**

***Core outcome measures in dementia studies and trials***

To understand current practice and general consensus regarding outcomes and outcome measures that are vital for collection in dementia trials (as part of a wider research project), firstly an existing relevant systematic review was detected^1^. While this review highlighted some key information regarding outcome measures, the search strategy was conducted between 2004-2014, and although quality of life outcome measures were only considered in ~13% of the studies, the authors noted that quality of life was more often reported in the more contemporary trials^1^, which could therefore be echoed if the search strategy were to be updated.

In an attempt to bridge the gap between the findings of this review and current practice, a search of dementia trials was conducted on the International Standard Randomised Controlled Trial Number (ISRCTN) registry database. The ISRCTN database includes study designs beyond randomised controlled trials alone, including observational and interventional trials^2^. The search involved solely the keyword “dementia”, and was then limited to completed and ongoing trials (no time period limit was applied). The findings of this search highlighted the difference in primary outcome used in trials by intervention type.

Following this, the literature was searched for core outcome sets. The purpose of core outcome set development is to define an agreed standarised set of outcomes that are recommended for inclusion when assessing interventions in trials. Generally, the process involves engaging various stakeholders, in the case of dementia for example, people with dementia, caregivers, healthcare professionals and policy makers. Due to the high degree of variability in outcome measures used in dementia trials, there has been recent interest in establishing core outcome sets for dementia studies. However due to the varying mechanisms of action of dementia interventions there is to date no singular core outcome set. Therefore core outcome sets for trials of the key dementia intervention types: *pharmacological^3^, psychosocial^4 5^* and *non-pharmacological^6 7^* interventions were identified and examined.

In addition, to gauge a policy perspective of dementia outcome measurement, and note which outcome measures have been recommended for collection – National Institute for Health and Care Excellence (NICE) guidelines and appraisal documents were consulted^8 9^, as well as the United States Food and Drug Administration (FDA) documentation^10^ and The International Consortium for Health Outcomes Measurement (ICHOM) recommendations^11^. It was the combination of these steps which resulted in the pre-defined core dementia outcome measures, summarised in Table 1 (within the main text).

1. Harrison JK, Noel-Storr AH, Demeyere N, et al. Outcomes measures in a decade of dementia and mild cognitive impairment trials. *Alzheimer's research & therapy* 2016;8(1):1-10.

2. BMC. ISRCTN registry 2021 [Available from: <https://www.isrctn.com/page/about> accessed 05/03/2021

3. Webster L, Groskreutz D, Grinbergs-Saull A, et al. Core outcome measures for interventions to prevent or slow the progress of dementia for people living with mild to moderate dementia: Systematic review and consensus recommendations. *PLoS One* 2017;12(6):e0179521.

4. Programme EJ. Dementia outcome measures: Charting new territory. European Union: JPND. 2015 [Available from: <https://www.neurodegenerationresearch.eu/> wp-content/uploads/2015/10/JPND-Report-Fountain.pdf

5. Moniz-Cook E, Vernooij-Dassen M, Woods R, et al. A European consensus on outcome measures for psychosocial intervention research in dementia care. *Aging and Mental Health* 2008;12(1):14-29.

6. Reilly ST, Harding AJ, Morbey H, et al. What is important to people with dementia living at home? A set of core outcome items for use in the evaluation of non-pharmacological community-based health and social care interventions. *Age and Ageing* 2020;49(4):664-71.

7. Harding AJ, Morbey H, Ahmed F, et al. What is important to people living with dementia?: the ‘long-list’of outcome items in the development of a core outcome set for use in the evaluation of non-pharmacological community-based health and social care interventions. *BMC geriatrics* 2019;19(1):1-12.

8. NICE. ARICEPT (DONEPEZIL) SUBMISSION TO THE NATIONAL INSTITUTE FOR HEALTH AND CLINICAL EXCELLENCE MULTIPLE TECHNOLOGY APPRAISAL (MTA), 2010.

9. Formulary BN. Dementia NICE BNF 2021 [Available from: <https://bnf.nice.org.uk/treatment-summary/dementia.html2021>.

10. (FDA) USFaDA. Center for Drug Evaluation and Research (CDER). Guidance for industry: Alzheimer’s disease: developing drugs for the treatment of early stage disease: Silver Spring, MD: U.S. Department of Health and Human Services; 2013 [Available from: <http://www.fda.gov/downloads/drugs/guidancecomplianceregulatory> information/guidances/ucm338287.pdf. Accessed 9 Aug 2016.

11. (ICHOM). TICfHOM. Dementia 2016 [Available from: <http://www.ichom.org/medical-conditions/dementia/>.
