# Supplementary material for: Convergent validity of EQ-5D with core outcomes in dementia: a systematic review
Source: Health Qual Life Outcomes. 2022 Nov 19;20:152. doi: 10.1186/s12955-022-02062-1 (PMC9675120; doi:10.1186/s12955-022-02062-1)
Supplement: Supplementary file 2 — Additional file 2. Complete search strategy. [file 12955_2022_2062_MOESM2_ESM.docx]

**Additional File 2**

**Complete search strategy**

| Run: 20/04/2021, Ovid MEDLINE ® (1996-April Week 2 2021) & APA PsycINFO (1987-April Week 2 2021) | | |
| --- | --- | --- |
| # | Searches | Results |
| 1 | (MoCA or Montreal Cognitive Assessment).mp | 6974 |
| 2 | (MMSE or mini mental state examination or mini-mental state examination).mp | 59159 |
| 3 | (ADAS-Cog or Alzheimer's Disease Assessment Scale-Cognitive Subscale).mp | 2059 |
| 4 | (SIB or severe impairment battery).mp | 6440 |
| 5 | (BADLS or Bristol activities of daily living scale).mp | 236 |
| 6 | (DAD or disability assessment for dementia scale).mp. | 7323 |
| 7 | (ADCS-ADL or Alzheimer's disease cooperative study ADL scale).mp. | 217 |
| 8 | Alzheimer's disease cooperative study activities of daily living scale.mp. | 103 |
| 9 | (Lawton scale or Lawton index or Lawton instrumental ADL scale or Lawton instrumental activities of daily living scale or Lawton IADL scale).mp. | 436 |
| 10 | (Katz ADL or Katz index or Katz index of independence in ADL or Katz index of independence in activities of daily living).mp. | 845 |
| 11 | (Barthel Index or Barthel scale or Barthel index for activities of daily living).mp | 8110 |
| 12 | (NPI or Neuropsychiatric Inventory or Neuropsychiatric inventory questionnaire).mp. | 7544 |
| 13 | (CMAI or cohen-mansfield agitation inventory or cohen mansfield agitation inventory).mp. | 939 |
| 14 | (CSDD or cornell scale for depression in dementia).mp. | 1144 |
| 15 | (GDS or geriatric depression scale).mp. | 15117 |
| 16 | (euro qual or euro qual5d or euro qol5d or eq-5d or eq5-d or eq5d or euroqual or euroqol or euroqual5d or euroqol5d or European QoL-5 dimensions or European quality of life 5 dimensions).mp. | 13752 |
| 17 | exp Dementia/ | 220550 |
| 18 | dement*.mp. | 171942 |
| 19 | (alzheimer* or AD).mp | 261540 |
| 20 | 17 or 18 or 19 | 375766 |
| 21 | 7 or 8 | 284 |
| 22 | 1 or 2 or 3 or 4 or 5 or 6 or 9 or 10 or 11 or 12 or 13 or 14 or 15 or 21 | 96986 |
| 23 | 16 and 20 and 22 | 228 |
| 24 | Limit 23 to English language | 218 |
| Additional search – CINAHL, terms:  Eq-5d or eq5d AND dementia or Alzheimer’s – limited to abstract, generated 66 references – limited to English language = 64 | | |
| Total hits = 284, 46 were duplicate = remaining 236 records | | |
