# Supplementary material for: Convergent validity of EQ-5D with core outcomes in dementia: a systematic review
Source: Health Qual Life Outcomes. 2022 Nov 19;20:152. doi: 10.1186/s12955-022-02062-1 (PMC9675120; doi:10.1186/s12955-022-02062-1)
Supplement: Supplementary file 3 — Additional file 3. [file 12955_2022_2062_MOESM3_ESM.docx]

**Additional File 3**

| **Inclusion** | **Exclusion** |
| --- | --- |
| Population with formal dementia diagnosis (any type of dementia) | Mild cognitive impairment alone |
| Must report one of the key outcome instruments AND EQ-5D | Non-English language |
| EQ-5D-5L or EQ-5D-3L | EQ-5D VAS alone |
| Self-report and/or proxy report EQ-5D | Caregiver studies (where outcomes were collected |
| Any study design i.e., RCT, observational | Previous systematic reviews (although chain search of references will be completed) |
| Contains extractable data | Grey literature |
|  | Conference abstracts |
|  | Protocols |
