# Supplementary material for: Convergent validity of EQ-5D with core outcomes in dementia: a systematic review
Source: Health Qual Life Outcomes. 2022 Nov 19;20:152. doi: 10.1186/s12955-022-02062-1 (PMC9675120; doi:10.1186/s12955-022-02062-1)
Supplement: Supplementary file 4 — Additional file 4. Quality assessment of included papers adapted from the GRADE assessment tool. [file 12955_2022_2062_MOESM4_ESM.docx]

Additional file 4; Quality assessment of included papers adapted from the GRADE assessment tool

|  | ***Population*** | ***Sample*** | | | ***Outcome assessment*** | ***Analysis*** | | | ***Data***  ***Limitations*** | **Quality score** |
| --- | --- | --- | --- | --- | --- | --- | --- | --- | --- | --- |
|  | **1. Is the study population clearly defined?** | **2. Are the sample characteristics clearly summarised?** | **3. What is the magnitude of the sample size? High>300, Medium 100-300, Low<100** | **4. Are sample groups clearly defined?** | **5. Are instrument administration details clear?** | **6. Are details of analyses provided?** | **7. Are relationships measured via statistical methods i.e., correlation, p value, 95% CI?** | **8.Are strength of association thresholds included within the study?** | **9.Have the authors noted or discussed missing data?** |  |
| **Ankri 2003** | Y | Y | 142 | Y | Y | Y | Y | Y | Y | HIGH |
| **Ashizawa 2021** | Y | Y | 287 | Y | Y | Y | Y | N | N | MEDIUM |
| **Bhattacharya 2010** | Y | Y | 321 | N/A | Y | Y | Y | N | N | MEDIUM |
| **Bonfiglio 2019** | Y | Y | 141 | Y | Y | Y | Y | N | N | MEDIUM |
| **Bostrom 2007** | Y | Y | 68 | Y | Y | Y | Y | N | Y | MEDIUM |
| **Bryan 2005** | Y | Y | 64 | Y | Y | Y | Y | Y | Y | HIGH |
| **Castro-Monteiro 2014** | Y | Y | 274 | Y | Y | Y | Y | N | Y | HIGH |
| **Diaz-Redondo 2014** | Y | Y | 545 | Y | N | Y | Y | Y | Y | HIGH |
| **Easton 2018** | Y | Y | 541 | Y | Y | Y | Y | Y | N | HIGH |
| **Ersek 2010** | Y | Y | 88 | Y | N | Y | Y | N | Y | MEDIUM |
| **Farina 2020** | Y | Y | 307 | Y | Y | Y | Y | N | Y | HIGH |
| **Garre-Olmo 2017** | Y | Y | 343 | Y | Y | Y | Y | N | N | HIGH |
| **Gonzalez-Velez 2015** | Y | Y | 412 | Y | N | Y | Y | N | N | MEDIUM |
| **Haaksma 2018** | Y | Y | 331 | N/A | Y | Y | Y | N | N | MEDIUM |
| **HeBmann 2016** | Y | Y | 395 | Y | Y | Y | Y | N | Y | HIGH |
| **Karlawish 2008 [1]** | Y | Y | 110 | Y | Y | Y | Y | Y | Y | HIGH |
| **Karlawish 2008 [2]** | Y | Y | 110 | Y | Y | Y | Y | Y | Y | HIGH |
| **King 2022** | Y | Y | 243 | Y | Y | Y | Y | N | Y | HIGH |
| **Kunz 2010** | Y | Y | 399 | Y | Y | Y | Y | Y | Y | HIGH |
| **Kuo 2010** | Y | Y | 140 | Y | Y | Y | Y | N | N | MEDIUM |
| **Martin 2019** | Y | Y | 1004 | N/A | Y | Y | Y | Y | Y | HIGH |
| **Michalowsky 2021** | Y | Y | 77 | Y | Y | Y | Y | Y | Y | HIGH |
| **Naglie 2011 [1]** | Y | Y | 370 | Y | Y | Y | Y | N | Y | HIGH |
| **Naglie 2011 [2]** | Y | Y | 412 | Y | Y | Y | Y | N | Y | HIGH |
| **Orgeta 2015** | Y | Y | 478 | Y | Y | Y | Y | Y | Y | HIGH |
| **Schiffczyk 2010** | Y | Y | 137 | Y | Y | Y | Y | N | N | MEDIUM |
| **Sheehan 2012** | Y | Y | 112 | Y | Y | Y | Y | N | Y | HIGH |
| **Trigg 2015** | Y | Y | 145 | Y | Y | Y | Y | N | N | MEDIUM |
| **van de Veek 2019** | Y | Y | 138 | Y | N | Y | Y | N | Y | MEDIUM |
| **Vogel 2006** | Y | Y | 48 | Y | Y | Y | Y | N | Y | MEDIUM |

a N/A – not applicable

Each question was scored 1 if Y, 0 if no or undetermined for all questions except question 2 where sample >300; 100- 300; <100 score 2, 1 and 0 respectively. The score was then calculated for the 9 questions = total up to 10 points, Can define 8-10 = high, 5-7 = medium, <5 = low
