# Supplementary material for: Convergent validity of EQ-5D with core outcomes in dementia: a systematic review
Source: Health Qual Life Outcomes. 2022 Nov 19;20:152. doi: 10.1186/s12955-022-02062-1 (PMC9675120; doi:10.1186/s12955-022-02062-1)
Supplement: Supplementary file 5 — Additional file 5. Summary of core outcome measures. [file 12955_2022_2062_MOESM5_ESM.docx]

| **Additional File 5**  ***Summary of core outcome measures*** | | | | | |
| --- | --- | --- | --- | --- | --- |
| Instrument name | Measure of | Estimated duration (mins) | Domains/ items | Format of administration | Scoring |
| MMSE | Cognition | 5 – 10 | Orientation to time, orientation to place, registration, attention and calculation, recall, language, repetition, complex commands | Questionnaire plus drawing task | 24-30, normal  19-23, MCI  10-18, moderate  <9, severe |
| ADAS-Cog | Cognition | 45 – 60 | Word recall, naming objects and fingers, following commands, constructional praxis, ideational praxis, orientation, word recognition task, remembering test directions, spoken language, comprehension, word-finding difficulty | Series of physical tasks | Range for 0 – 70, >18 indicating greater cognitive impairment |
| Katz ADL | Function | 10 – 15 | Bathing, dressing, toileting, transferring, continence, feeding | Observation or interview | 0 – 6: 6, full function, 4, moderate impairment, <2, severe functional impairment |
| ADCS-ADL | Function | 15 – 30 | Personal belongings, selecting clothes, dressing, cleaning, handle finances, write things, laundry, keep appointments, use telephone, prepare food, get around, discuss current events, read, watch television, go shopping, left alone, use household appliances, hobbies | Informant-based questionnaire | 0 – 78, lower scores indicate greater severity |
| Barthel Index | Function | 20 | Bowels, bladder, grooming, toilet use, feeding, transfer, mobility, dressing, stairs, bathing | Medical records, direct observation or interview | 0 – 100, totally dependent – completely independent |
| Lawton Scale | Function | 10 – 15 | Use telephone, shopping, prepare food, housekeeping, laundry, mode of transportation, medication responsibility, handle finances | Interview based questionnaire | 0 – 8, low function – high function |
| DAD | Function | 15 – 20 | Hygiene, dressing, continence, eating, meal preparation, telephoning, outings, finance and correspondence, medications, leisure and housework | Interview-based informant questionnaire | 0 – 100, most severe – without disabilities |
| BADLS | Function | Unclear | Preparing food, eating, preparing drink, drinking, hygiene, teeth, bath/shower, toilet/commode, transfers, mobility, orientation – time, orientation – space, communication, telephone, housework/gardening, shopping, finances, games/hobbies, transport | Informant-based questionnaire | 0 – 60, totally independent – totally dependent |
| NPI | Behaviour/mood: neuropsychiatric symptoms | 20 – 30 | Delusions, hallucinations, agitation/aggression, dysphoria, anxiety, euphoria, apathy, disinhibition, irritability/lability, aberrant motor activity | Interview-based informant questionnaire | 0 – 144, higher scores indicate greater severity |
| CSDD | Behaviour/mood: depression | 20 | Mood related signs, behavioural disturbance, physical signs, cyclic functions, ideational disturbance | Interview-based informant questionnaire | 0 – 38, higher scores indicate greater severity, >12 indicates probable depression |
| GDS | Behaviour/mood: depression | <10 | Life satisfaction, activities/ interests, emptiness, boredom, good spirits, fear, happiness, helplessness, staying at home or going out, memory problems, wonderful to be alive, worthlessness, energy, hopelessness, better off | Interview based questionnaire | 0 – 15; 0-4, normal, 5-8, mild depression, 9-11, moderate depression, 12-15 severe depression |
| CMAI | Behaviour/mood: agitation | 20 | Hitting, kicking, grabbing, pushing, throwing, biting, scratching, spitting, hurting, destroying, physical advances, pacing, disrobing, different place, falling, inappropriate eating/ drinking, inappropriate handling, hiding things, hoarding, repetitious mannerisms, restlessness, screaming, verbal advances, cursing/ verbal aggression, repetitive questions, strange noises, complaining, negativism, unwarranted requests for help | Informant-based questionnaire | 29 – 203, higher scores indicate greater severity |
