# Supplementary material for: Convergent validity of EQ-5D with core outcomes in dementia: a systematic review
Source: Health Qual Life Outcomes. 2022 Nov 19;20:152. doi: 10.1186/s12955-022-02062-1 (PMC9675120; doi:10.1186/s12955-022-02062-1)
Supplement: Supplementary file 6 — Additional file 6. Evidence of EQ-5D convergent validity with cognitive measures. [file 12955_2022_2062_MOESM6_ESM.docx]

| **Additional File 6**  ***Evidence of EQ-5D convergent validity with cognitive measures*** | | | | | |
| --- | --- | --- | --- | --- | --- |
| Study reference | Cognition measure | Evidence of association | Regression analysis (y/n) | Regression details and results | Dimension specific evidence |
| Ankri et al | MMSE | Anxiety/ depression EQ-5D dimension associated with MMSE score; moderately anxious/ depressed subjects have higher MMSE scores (F=6.86, p=0.001) (proxy rated) | N | n/a | MMSE score was only associated with anxiety/ depression dimension |
| Ashizawa et al | MMSE | MMSE was grouped into severity stages of mild, moderate, and severe. Z statistic = 9.22, p<0.001. EQ-5D was significantly lower in severe dementia | Y | Multiple linear regression used to identify factors impacting EQ-5D-5L proxy ratings: MMSE was not a significant determinant in the multiple regression model. Coefficient = -0.00533, p=0.883 | n/a |
| Bhattacharya et al | MMSE | Neither proxy (r=-0.025) nor self (r=-0.087) reported EQ-5D correlated with MMSE (p>0.001) | N | n/a | n/a |
| Bonfiglio et al | MMSE | No significant correlation with self (r=-0.157) or proxy reported (r=-0.113) EQ-5D index scores (p>0.05) | Y | No MMSE evidence reported | No MMSE evidence reported |
| Bonfiglio et al | ADAS-Cog | Self-rated EQ-5D index score correlation with ADAS-cog (Rho=0.173, p=0.041), but proxy rated scores did not (Rho=-0.160, p=0.188) | Y | No ADAS-Cog evidence reported | No MMSE evidence reported |
| Bostrom et al | MMSE | No MMSE evidence reported | Y | No MMSE evidence reported | n/a |
| Bryan et al | MMSE | No MMSE evidence reported | N | n/a | No MMSE evidence reported |
| Castro-Monteiro et al | MMSE | No MMSE evidence reported | Y | No MMSE evidence reported | No MMSE evidence reported |
| Diaz-Redondo et al | MMSE | No MMSE evidence reported | Y | No MMSE evidence reported | No MMSE evidence reported |
| Ersek et al | MMSE | EQ-5D scores (proxy rated) correlated significantly with MMSE score (Rho=0.361, p=0.01) | N | n/a | n/a |
| Farina et al | MMSE | Reported in regression | Y | MMSE was significantly associated with EQ-5D proxy scores in both the uncontrolled (β = 0.42, p < 0.001) and controlled model (β = 0.23, ΔR^2^ = 0.04, p < 0.001), however was not associated with self-rated EQ-5D in the uncontrolled model (β = − 0.02, p = 0.83), or controlled model (β = − 0.04, ΔR^2^ = 0.001, p = 0.63) | n/a |
| Garre-Olmo et al | MMSE | MMSE was not significantly correlated to proxy EQ-5D score at any CDR defined severity: 1, r=0.110; 2, r=0.139; 3, r=0.080 (p>0.05) | Y | No MMSE evidence reported | n/a |
| Gonzalez-Velez et al | MMSE | No MMSE evidence reported | Y | No MMSE evidence reported | n/a |
| Haaksma et al | MMSE | EQ-5D (proxy) does not correlate with MMSE (r=0.029, p=0.61) | N | n/a | n/a |
| Heßmann et al | MMSE | EQ-5D scores decreased with increasing cognitive impairment (as defined by MMSE). This change was not significant for self-ratings, but was for proxy-ratings: 0-9, 0.21+/-0.27; 10-19, 0.41+/-0.34; 20-26, 0.61+-/-0.33; >26, 0.75+/-0.30; p<0.001 | Y | Multivariate linear regression showed that self-reported EQ-5D index was considerably correlated with diminished cognitive capacity (but not for proxy scores) (β =-0.010, p<0.05) | Severely demented (MMSE 0-9) reported considerably fewer problems in almost all dimensions vs. less cognitively impaired patients |
| Karlawish et al (1) | MMSE | No statistically significant differences were found between self-rated EQ-5D index scores at different MMSE defined severities: 24-29, 0.780; 20-23, 0.800; 11-19, 0.885 (p=0.16) | Y | No MMSE evidence reported | A notable proportion of patients did not report disability in domains where one might reasonably expect disability, specifically the usual activities item |
| Karlawish et al (2) | MMSE | No statistically significant differences were found between proxy-rated EQ-5D index scores at different MMSE defined severities: 24-29, 0.720; 20-23, 0.630; 11-19, 0.604 p=0.13) | Y | In least-squares regression, MMSE score did not predict EQ-5D scores | n/a |
| King et al | MMSE | EQ-5D proxy was significantly associated with severe cognitive impairment as measured by the MMSE for both regression models, but not for EQ-5D self-report. Model 1 = -0.16, p<0.01; Model 2 = 0,29, p<0.01 | Y | Linear regression, results in previous box | n/a |
| Kunz et al | MMSE | Weak correlation between MMSE with EQ-5D (Pearson correlation coefficients and 95% confidence intervals: self-rated EQ-5D, 0.18 [0.08-0.27] and proxy rated EQ-5D, 0.24 [0.14-0.34] | Y | No MMSE evidence reported | No MMSE evidence reported |
| Kuo et al | MMSE | Reported in regression | Y | Multiple regression model showed that cognitive impairment stages, as classified by MMSE, show no significant determinant of QOL (p>0.001): mild-to-moderate, β -0.038, t -0.72; moderate, β -0.032, t -0.51; severe, β -0.074, t -1.1 | n/a |
| Michalowsky et al | MMSE | Neither EQ-5D-5L or EQ-5D-3L were able to discriminate between of cognitive impairment 3L p = 0.267, 5L p = 0.057 | N | n/a | Y – but no results related to cognition |
| Naglie et al (1) | MMSE | No significant relationship between mean EQ-5D (self-rated) scores and MMSE scores [results not shown] (p>0.05) | Y | MMSE scores (results not shown) were not significant independent predictors of EQ-5D ratings | n/a |
| Naglie et al (1) | ADAS-Cog | No significant relationship between mean EQ-5D (self-rated) scores and ADAS-Cog [results not shown] | Y | ADAS-cog scores (results not shown) were not significant independent predictors of EQ-5D ratings | n/a |
| Naglie et al (2) | MMSE | Mean EQ-5D ratings were consistently lower with more severe cognitive impairment (MMSE results not shown) | Y | No MMSE evidence reported | n/a |
| Naglie et al (2) | ADAS-Cog | Increasing cognitive impairment – as defined by ADAS-Cog scores, resulted in lower mean EQ-5D: 8-21, 0.82 (0.16); 22-25, 0.80 (0.16); 26-34, 0.76 (0.17); 35-70, 0.68 (0.22). All of which are significant at p<0.0001 | Y | Multiple linear regression showed that ADAS-Cog was not a significant predictor of EQ-5D (proxy-rated) scores: B -0.020, p=0.070, R^2^=0.05 | n/a |
| Schiffczyk et al | MMSE | MMSE severity was not significantly associated with patient-self ratings, p=0.148; but was significantly associated with proxy ratings of patient, p=0.002 | N | n/a | n/a |
| Schiffczyk et al | ADAS-Cog | No ADAS-Cog evidence reported | N | n/a | n/a |
| Trigg et al | MMSE | No significant association between MMSE change and EQ-5D change (r=0.02) or EQ-5D proxy change (r=0.22), p>0.01 (exact p values not reported) | Y | For changes in EQ-5D proxy ratings, MMSE change scores were significant explanatory variables- improvement in cognition was associated with larger increases in proxy utility ratings (p<0.05) | n/a |
| van de Beek et al | MMSE | Reported in regression | Y | Linear mixed models show that MMSE has no significant association with (self-rated) EQ-5D utility. Model 1 – B 0.06 (0.27) (p>0.05) | n/a |
| Vogel et al | MMSE | No MMSE evidence reported | N | n/a | No MMSE evidence reported |
