# Supplementary material for: Convergent validity of EQ-5D with core outcomes in dementia: a systematic review
Source: Health Qual Life Outcomes. 2022 Nov 19;20:152. doi: 10.1186/s12955-022-02062-1 (PMC9675120; doi:10.1186/s12955-022-02062-1)
Supplement: Supplementary file 7 — Additional file 7. Evidence of EQ-5D convergent validity with function measures. [file 12955_2022_2062_MOESM7_ESM.docx]

| \| **Additional File 7**  ***Evidence of EQ-5D convergent validity with function measures*** \| \| --- \| | | | | | |
| --- | --- | --- | --- | --- | --- | --- |
| Study reference | ADL measure | Evidence of correlation | Regression analysis (y/n) | Regression details and results | Dimension specific evidence |
| Ankri et al | Katz | Correlation between Katz and mobility (F = 16.4, P < .0001), self-care (F = 6.3, P < .0001), usual activities (F = 6.8, P <.002), and pain (F = 6.8, P <.002) | N | n/a | Katz correlates with mobility, self-care, usual activities and pain/discomfort – but not anxiety/ depression |
| Ashizawa et al | Barthel index | BI was the only significant determinant in the multiple regression analysis - coefficient 0.880, p<0.001 | Y | Multiple regression analysis used to identify factors impacting EQ-5D-5L proxy ratings, results in previous box | n/a |
| Bhattacharya et al | ADCS-ADL | ADCS-ADL and EQ-5D index score correlation: proxy, r=0.284, p<0.001; self, r=0.061, p>0.001 | N | n/a | n/a |
| Bonfiglio et al | Barthel index | Barthel index and EQ-5D index score correlation: proxy, r=0.470, p=0.000; self, r=0.203, p=0.016 | Y | Multivariate linear regression analyses showed that Barthel ADL was a significant independent factor affecting patient QoL. In both models, BADL was a significant predictor of self (model 1: p=0.018, model 2: p=0.008) and proxy rated EQ-5D scores (model 1: p=0.008, model 2: p=0.023) | No Barthel index evidence reported |
| Bonfiglio et al | Lawton index | Lawton index and EQ-5D index score correlation: proxy, r=0.339, p=0.005; self, r=-0.054, p=0.531 | Y | No Lawton index evidence reported | No Lawton index evidence reported |
| Bostrom et al | DAD | Reported in regression | Y | Linear regression analyses showed that independency in IADL was a significant determinant of (proxy rated) EQ-5D (β, 0.019, p=0.015) | n/a |
| Bryan et al | BADLS | At a group level, proxies describe patients as having poorer overall EQ-5D where there are greater limitations in ADL (assessed by BADLS) | N | n/a | EQ-5D mobility and self-care (proxy rated) dimensions show positive association with BADLS summary scores – association p<0.01 |
| Castro-Monteiro et al | Barthel index | Reported in regression | Y | In multiple linear regression models the Barthel Index was shown to have a significant association with EQ-5D (proxy) index scores (β =0.35; p<0.001) | n/a |
| Diaz-Redondo et al | Barthel index | EQ-5D (proxy) index scores were significantly lower for people with lower Barthel index | Y | In multiple linear regression, the Barthel index was a significant determinant for EQ-5D (proxy) index (β=0.70, p<0.05) | Reporting problems (as opposed to no problems) in all EQ-5D dimensions except for anxiety/depression was significantly associated with Barthel index score p<0.01 |
| Easton et al | Barthel index | Barthel index and EQ-5D index scores correlation: proxy, r=0.560, p=0.01; self, r=0.492, p=0.01 | N | n/a | Barthel index was correlated with EQ-5D self-rated dimensions: mobility, -0.499, self-care, -0.609, usual activities, -0.374; and proxy rated dimensions: mobility, -0.555, self-care, -0.627, usual activities, -0.577. All p<0.01 |
| Garre-Olmo et al | DAD | DAD was significantly correlated to proxy EQ-5D score at all CDR defined severity: 1, r=0.264; 2, r=0.400; 3, r=0.450 (p<0.05) | Y | No DAD evidence reported | n/a |
| Gonzalez-Velez et al | Barthel | EQ-5D (proxy) index scores were 0.5 points greater in those with moderate (>40) vs. severe (<40) dependency defined with Barthel index scores (p<0.001) | Y | No Barthel index evidence reported | n/a |
| Haaksma et al | DAD | EQ-5D (proxy) was significantly correlated with increased daily functioning (r=0.302, p<0.0001). One SD increase in EQ-5D was associated with an average 2.97% increase in DAD score | N | n/a | n/a |
| Heßmann et al | ADCS-ADL | Total ADCS-ADL score was correlated with self (r=0.241, p<0.001) and proxy (r=0.682, p<0.001) EQ-5D index scores in bivariate analyses | Y | Multivariate regression analysis found that ADCS-ADL was a significant predictor of proxy rated EQ-5D (β=0.012, p<0.05), but not self-rated (β=0.002, p>0.05) | No ADCS-ADL evidence reported |
| Karlawish et al (1) | Lawton scale | Correlation between lower EQ-5D (self) scores and greater IADL impairment (p=0.03). Self-rated EQ-5D scores were associated with BADL insight (p=0.05), but not IADL insight (p=0.11) | Y | Regression models showed that greater awareness of BADL deficits is an independent predictor of lower EQ-5D | 41% of the EQ-5D were a 1.0, which represents perfect health. Participants did not report disability in domains where one might reasonably expect disability – specifically the EQ-5D item usual activities |
| Karlawish et al (2) | Lawton scale | Greater IADL and BADL deficits were associated with decline in proxy EQ-5D ratings (p=0.0000). | Y | Least-squares regression showed that proxy ratings of PwD BADLs remained a strong independent predictor of EQ-5D scores (β=-0.023, p=0.000) | n/a |
| King et al | BADLS | EQ-5D proxy was significantly associated with level of disability as measured by BADLS for both regression models, but not for EQ-5D self-report. Model 1 = -0.25, p<0.01; Model 2 = -0.20, p<0.01 | Y | Linear regression, results reported in previous box | n/a |
| Kunz et al | Barthel index | EQ-5D self and proxy correlated with Barthel index (r=0.50 [0.41-0.57], r=0.67 [0.61-0.72] respectively, as the confidence intervals do not overlap – indicated statistically significant difference | Y | Generalised linear regression explored factor impact on inter-rater agreement | No Barthel index evidence reported |
| Kuo et al | Barthel index | Reported in regression | Y | Multiple regression models showed that dependence, as defined by Barthel index scores, was a significant determinant of (self-rated) QoL (p<0.001): medium, β=-0.119, t=-2.28; high, β=-0.441, t=7*, low, β=-0.629, t=8.36*, p<0.001* | n/a |
| Naglie et al (1) | DAD | No significant relationship between mean EQ-5D (self) ratings and DAD scores (p>0.05), no correlation reported | Y | Multiple linear regression showed that DAD score was not a significant predictor of EQ-5D (self) ratings (p>0.05) | n/a |
| Naglie et al (2) | DAD | With increasing function threshold brackets, as defined by DAD, there were significantly greater mean EQ-5D (proxy) scores (p<0.0001): 0-49, 0.65 (0.20); 50-68, 0.76 (0.14); 69-88, 0.79 (0.18); 89-100, 0.85 (0.17) | Y | Multiple linear regression showed that DAD score was a consistent significant independent predictor of EQ-5D (proxy) scores; β=0.051, p<0.0001, R^2^=0.09 | n/a |
| Orgeta et al | BADLS | Reported in regression | Y | Multiple linear regression showed that BADLS was an independent predictor of both self: β=-0.257, and proxy: β=-0.463 (p<0.01) EQ-5D index scores | No BADLS evidence reported |
| Sheehan et al | Lawton scale | Reported in regression | Y | Linear regression models showed that Lawton score was a significant predicter of EQ-5D proxy (0.007 (0.028, 0.126) p=0.0034), but not self-rated EQ-5D (-0.014 (-0.059, 0.030) p=0.5316) | n/a |
| Trigg et al | DAD | No significant association between changes on DAD and EQ-5D self or proxy – r=0.06, r=0.13, p>0.05 respectively | Y | Multiple linear regression showed that DAD change did not account for EQ-5D (self) scores | n/a |
| van de Beek et al | DAD | Lower EQ-5D (self) scores were associated with lower DAD scores ±SE = 0.2 ± 0.1, p < 0.05 | Y | In multivariate models with backward selection, DAD remained an independent determinant of EQ-5D (self) score (±SE=0.1±0.1, p<0.05) | n/a |
