# Supplementary material for: Convergent validity of EQ-5D with core outcomes in dementia: a systematic review
Source: Health Qual Life Outcomes. 2022 Nov 19;20:152. doi: 10.1186/s12955-022-02062-1 (PMC9675120; doi:10.1186/s12955-022-02062-1)
Supplement: Supplementary file 8 — Additional file 8. Evidence of EQ-5D convergent validity with behaviour measures. [file 12955_2022_2062_MOESM8_ESM.docx]

| **Additional File 8**  ***Evidence of EQ-5D convergent validity with behaviour measures*** | | | | | |
| --- | --- | --- | --- | --- | --- |
| Study reference | Behaviour/ mood measure | Evidence of correlation | Regression analysis (y/n) | Regression details and results | Dimension specific evidence |
| Bhattacharya et al | NPI | NPI-Q displayed negative correlations EQ-5D: self, r=-0.144, p>0.001; proxy, r=-0.385, p<0.001 | N | n/a | n/a |
| Bhattacharya et al | CSDD | CSDD displayed negative correlations with EQ-5D: self, r=-0.297, p<0.001; proxy, r=-0.308, p<0.001 | N | n/a | n/a |
| Bonfiglio et al | GDS | GDS showed negative correlations with EQ-5D: self, r=-0.279, p=0.000; proxy, r=-0.132, p=0.279 | Y | Multivariate linear regression analyses showed GDS was a significant predictor of self-rated EQ-5D (β=-0.182, p=0.042) | No GDS evidence reported |
| Bostrom et al | NPI | Reported in regression | Y | Linear regression analyses showed that NPI was a significant determinant of (proxy rated) EQ-5D (β=-0.008, p=0.003) | n/a |
| Bryan et al | NPI | At a group level, proxies describe patients as having poorer overall EQ-5D where there is a higher presence of behavioural and psychological symptoms (assessed by NPI) | N | n/a | Higher NPI scores are seen (on average) in level 2 than level 1 anxiety/depression item rating |
| Castro-Monteiro et al | CSDD | No CSDD evidence reported | Y | No CSDD evidence reported | n/a |
| Diaz-Redondo et al | CSDD | There was no statistically significant difference in EQ-5D index (proxy) score between known groups of depression (CSDD <6 vs. CSDD >6) | Y | No CSDD evidence reported | Reporting problems in the anxiety/depression dimension was significantly associated with CSDD defined known groups (p<0.01) |
| Easton et al | NPI | NPI showed a negative correlation with EQ-5D self, r=-0.099; but a positive correlation with EQ-5D proxy, r=0.056 or proxy ratings. P>0.05 for both | N | n/a | Proxy rated EQ-5D dimensions: mobility and anxiety/depression show significant association with NPI (-0.177, 0.160 <0.01 respectively). No significant associations found with self-reported EQ-5D dimensions |
| Farina et al | NPI | Reported in regression | Y | Within the regression model having neuropsychiatric symptoms (β =−0.26, p < 0.00) was significantly associated with poor EQ-5D proxy scores, but no significance with self-rated EQ-5D (β =−0.13, p < 0.09) | n/a |
| Garre-Olmo et al | NPI | A negative correlation was found between NPI and EQ-5D at all dementia stages, however this was only statistically significant in mild dementia (r=-0.135, p<0.05) | Y | No NPI evidence reported | n/a |
| Gonzalez-Velez et al | CSDD | There was no statistically significant difference in EQ-5D index (proxy) score between known groups of depression (CSDD <6 vs. CSDD >6), p>0.05 | Y | No CSDD evidence reported | n/a |
| Haaksma et al | NPI | EQ-5D (proxy) was significantly correlated with fewer neuropsychiatric symptoms (r=-0.258, p<0.0001). One SD increase in EQ-5D was associated with a 2.41% point decrease in NPI | N | n/a | n/a |
| Heßmann et al | NPI | Negative correlation seen between NPI scores and EQ-5D index scores: proxy, r=-0.492, p<0.01; self, r=-0.064, p=0.476 | Y | Multivariate regression analysis found that NPI was associated with proxy rated EQ-5D (β=-0.008, p=0.002) | No NPI evidence reported |
| Heßmann et al | GDS | Negative correlation between mean GDS score and EQ-5D index scores: self, r=-0.557, p<0.01; proxy, r=-0.295, p<0.01 | Y | Multivariate regression analysis found that GDS was associated with EQ-5D self-score (β=-0.025, p<0.05) | No GDS evidence reported |
| Karlawish et al (1) | GDS | Mean EQ-5D (self) decreased with increasing GDS score: 0-1, 0.886; 2-3, 0.872; 4-11, 0.695; p=0.0004 | Y | No GDS evidence reported for EQ-5D prediction | No GDS evidence reported |
| Karlawish et al (2) | GDS | GDS evidence related to caregiver | Y | No GDS evidence reported | n/a |
| Martin et al | CMAI | A statistically significant relationship was observed between the self-rated EQ-5D-5L and CMAI (rho=-0.0663, p<0.05) (not for proxy rated) however the effect size was very small (8-unit increase in CMAI associated with 0.01 reduction in utility) | Y | The relationship between the proxy EQ-5D-5L utility scores and CMAI was statistically significant in regression results (empirical data not reported) | n/a |
| Michalowsky et al | GDS | Correlation coefficients of >0.3 were considered to indicate moderate convergent validity. GDS showed a greater degree of correlation with index scores of EQ-5D-3L (co= -0.372) than index scores than 5L (co= -0.311). In addition, both EQ-5D-5L and EQ-5D-3L were able to discriminate between groups of GDS depression states, p=0.001 for both measures | N | n/a | GDS correlation coefficient with mobility (3L) = 0.310; self-care (3L) = 0.446; UA (3L) = 0.414; poor correlation with pain/discomfort; anxiety/depression (5L) = 0.317. |
| Naglie et al (1) | GDS | With increasing GDS scores, there were significantly lower mean EQ-5D (self) ratings: 0-4, 0.94; 5-10, 0.88; 11-13, 0.80; 14-30, 0.70 (p<0.0001) | Y | Multiple linear regression showed that GDS score was the only consistent significant independent predictor of self-rated EQ-5D (p<0.0001) | n/a |
| Naglie et al (1) | NPI | With increasing NPI scores, there were lower EQ-5D mean scores, however they were not statistically significantly different p>0.05 | Y | Multiple linear regression found that NPI was not a significant predictor of EQ-5D (self) scores | n/a |
| Naglie et al (2) | GDS | With increasing GDS scores, there were significantly lower mean EQ-5D (proxy) ratings: 0-10, 0.84; 11-13, 0.77; 14-20, 0.72; 21-30, 0.65 (p<0.0001) | Y | Multiple linear regression showed that GDS was a consistent statistically significant independent predictor of proxy rated EQ-5D (p<0.0001) | n/a |
| Naglie et al (2) | NPI | With increasing NPI scores, there were significantly lower mean EQ-5D (proxy) scores: 0, 0.84; 1-6, 0.81; 7-14, 0.75; 15-73, 0.68 (p<0.0001) | Y | NPI was not a statistically significant predictor of EQ-5D (proxy) scores | n/a |
| Orgeta et al | CSDD | Reported in regression | Y | Multivariate linear regression showed that CSDD was not a significant predictor of self-rated EQ-5D (β=-0.303), but was for proxy rated EQ-5D (β=-0.132) p<0.05 | No CSDD evidence reported |
| Schiffczyk et al | GDS | No GDS evidence reported | Y | n/a | n/a |
| Sheehan et al | GDS | GDS had negative correlation with EQ-5D index scores: self, r=-0.016, p=0.0814; proxy, r=-0.004, p=0.6868 | Y | Linear regression models showed that GDS was not a predictor of EQ-5D self β=-0.016, p=0.0814; or proxy β=-0.004, p=0.6868 | n/a |
| Trigg et al | NPI | No significant association between changes on NPI and changes on EQ-5D: self, r=0.09 or proxy r=-0.05; p>0.05 | Y | Multiple linear regression showed that NPI change did not account for EQ-5D (self) scores | n/a |
| van de Beek et al | GDS | Reported in regression | Y | In multivariate models with backward selection, GDS remained an independent determinant of EQ-5D (self) score (±SE = -3.7 ± 0.4, p < 0.001) | n/a |
| van de Beek et al | NPI | NPI subscales only: presence of depression and anxiety on NPI were associated with lower EQ-5D (self) scores (depression ±SE =-5.6 ± 2.5, p<0.05; anxiety ±SE =-5.4±2.6, p<0.05) | Y | Multivariate models showed that NPI was not a significant predictor of EQ-5D (self) scores | n/a |
| Vogel et al | GDS | No GDS evidence reported | no | n/a | n/a |
